# Assessment of Neuropathic Pain in Erosive Hand Osteoarthritis

**DOI:** 10.3390/jcm13113244

**Published:** 2024-05-31

**Authors:** Marta Favero, Mario Cacciavillani, Francesca Ometto, Mariagrazia Lorenzin, Giacomo Cozzi, Laura Scagnellato, Stefania Vio, Andrea Doria, Chiara Briani, Roberta Ramonda

**Affiliations:** 1 Rheumatology Unit, Department of Medicine-DIMED, Padova University Hospital, 35128 Padova, Italy; faveromarta@gmail.com (M.F.); mariagrazialorenzin@libero.it (M.L.); giacomo.cozzi@aopd.veneto.it (G.C.); laurascagnellato@gmail.com (L.S.); adoria@unipd.it (A.D.); 2 Internal Medicine I, Cà Foncello Hospital, 31100 Treviso, Italy; 3 Specialistic Medical Center (CEMES), EMG Laboratory, Synlab Data Medica, 35128 Padova, Italy; mario.cacciavillani@gmail.com; 4 ULSS 6 Euganea Company, 35131 Padova, Italy; f.ometto@gmail.com; 5 Radiology Unit, Padova University Hospital, 35128 Padova, Italy; stefania.vio@aopd.veneto.it; 6 Department of Neurosciences, Neurology Unit, University Hospital of Padova, 35128 Padova, Italy; chiara.briani@unipd.it

**Keywords:** erosive hand osteoarthritis, neuropathic pain, nociplastic pain, carpal tunnel syndrome

## Abstract

**Background/Objectives:** Erosive hand osteoarthritis (EHOA) is an aggressive form of hand osteoarthritis (OA) and a severely disabling condition. Patients affected by OA frequently lament symptoms suggestive of neuropathic pain (NP). The aim of our study was to ascertain the presence and severity of NP in patients with EHOA and correlate its presence with EHOA clinical characteristics. **Methods:** In this retrospective study, we included all consecutive EHOA patients with NP symptoms who underwent upper limb electroneurography (ENoG) and nerve ultrasound. The presence of NP was screened using the ID pain neuropathic pain-screening questionnaire (ID-Pain). In addition, the following NP questionnaires were also used: Douleur Neuropathique en 4 Questions (DN4), PainDETECT, and Neuropathic Pain Symptom Inventory (NPSI). Moreover, patients completed the Australian/Canadian Osteoarthritis Hand Index (AUSCAN) and Dreiser’s algofunctional finger index questionnaires assessing EHOA disease activity. The following clinical and laboratory data were collected: age, sex, BMI, disease duration, intensity of pain (VAS 0–10), painful and swollen joints, and inflammatory indices, as well as C-reactive protein (CRP) and erythrocyte sedimentation rate (ESR). **Results:** Of the 34 patients studied, 24 (70.6%) presented NP to the ID-Pain questionnaire. According to DN4, 14 (41.2%) patients had NP, while using the PainDETECT questionnaire, 67.6% had NP. Patients with NP were statistically younger and had a higher VAS pain score compared to subjects without NP. The ENoG and median nerve ultrasound were normal in 81% of patients, while four patients had carpal tunnel syndrome. The ID-Pain questionnaire correlated with the number of painful joints (r = 0.48, *p* = 0.03) and with the AUSCAN questionnaire (r = 0.37, *p* = 0.05). The DN4 questionnaire correlated with PainDETECT (r = 0.58, *p* < 0.01). The PainDETECT questionnaire correlated with VAS pain (r = 0.49, *p* = 0.02), the DN4 questionnaire (r = 0.58, *p* < 0.01), and AUSCAN (r = 0.51, *p* = 0.02). The NPSI questionnaire correlated negatively with BMI (r = −0.53, *p* = 0.01) and positively with the PainDETECT questionnaire (r = 0.49, *p* = 0.02). **Conclusions:** Our study revealed that 32% to 70% of EHOA patients exhibited symptoms consistent with NP, with observed variability depending on the questionnaire utilized. Despite patients frequently exhibiting symptoms compatible with NP, only 19% of patients presented alterations on ENoG and ultrasound examinations confirming CTS. This suggests a probable nociplastic component for pain in patients with EHOA, which warrants tailored treatment. In the present study, NP correlated with clinical and functional indices of EHOA.

## 1. Introduction

Erosive hand osteoarthritis (EHOA) is an aggressive form of hand osteoarthritis (OA) and a severely disabling condition that results in pain, functional limitation, and reduced quality of life [1]. Although more severe than inflammatory arthritis and non-erosive hand OA [2], available treatments capable of modifying the natural course of EHOA are scarce [1,3]. Most clinical trials with conventional immunosuppressants or biologic drugs have not yielded the expected results [4,5]. A recent trial with subcutaneous denosumab 60 mg every 3 months showed some promising results in EHOA patients, demonstrating efficacy against disease progression, occurrence of new erosions, and painful symptoms measured by the visual analogue scale (VAS) and functional indices [6]. Nevertheless, it remains of the utmost importance to study the pathogenetic mechanisms underlying EHOA to identify novel potential therapeutic targets.

Generally speaking, pain can be classified as nociceptive, neuropathic, or nociplastic. Patients affected by OA frequently lament symptoms suggestive of neuropathic pain (NP), such as tingling, burning, and pins and needles sensations. In subjects affected by knee OA, NP correlates with the severity of symptoms, joint function, psychological well-being, and the intake of painkillers [7,8]. Although understudied, it has been reported that NP may occur in 16–42% of patients with hand OA using different assessment questionnaires [9,10]. In the Magni N et al. cohort of 121 patients with symptomatic hand OA, the prevalence of NP was estimated at 42% using the Douleur Neuropathique en 4 Questions (DN4) questionnaire [9]. Conversely, in a study comprising 91 patients using the PainDETECT instrument, 16% were deemed likely to have NP, 53% undetermined, and 31% not likely [10]. In this study, 73% of patients had EHOA, defined as having at least one proximal (PIP) or distal interphalangeal joint (DIP) in the erosive or remodeling phase according to the Verbruggen–Veys score [10]. The frequency of NP was higher in younger subjects and was associated with less radiographic damage and a lower Short Form Health Survey 36 (SF-36) physical component summary score at baseline. The presence of NP positively correlated with female sex, VAS pain (inclusion criteria VAS ≥ 3) and comorbidities measured by the self-administered comorbidity questionnaire. Prednisolone treatment did not alleviate NP symptoms. On the other hand, the presence of NP did not affect the efficacy of prednisolone against inflammatory pain [10]. In addition, lower pressure pain threshold (PPT)—indicating pain sensitization, measured at the most painful interphalangeal finger joint with a digital handheld algometer—has been associated with more tender and painful hand joints in a cohort of 206 patients affected by hand OA (mean age 61 (57–66) years, mean BMI 26.4, median disease duration 6 years) [11].

Furthermore, concomitant carpal tunnel syndrome (CTS) has been observed in approximately 9% of symptomatic hand OA patients, with a higher prevalence in individuals with obesity, metabolic syndrome, and hypothyroidism [12]. However, there is conflicting evidence as it relates to the association between CTS and symptoms as well as the number of painful joints in hand OA [12,13]. All these findings underscore the multifaceted nature of pain in hand OA, necessitating a comprehensive understanding of its diverse etiological factors to devise targeted therapeutic interventions [14].

The primary objective of our study was to ascertain the presence and severity of NP in patients with EHOA using patient-reported questionnaires. The secondary objective was to assess the agreement between the different questionnaires used to evaluate the presence of NP. Moreover, we also aimed to determine whether patients with NP presented with electroneurography (ENoG) or median nerve ultrasound abnormalities. Finally, we endeavored to investigate the association between NP and the peculiar clinical characteristics of EHOA.

We hypothesize that NP in EHOA is frequently encountered and correlates with disease indices. However, some of the neuropathic symptoms reported by patients with EHOA might be attributable to carpal tunnel syndrome.

## 2. Materials and Methods

### 2.1. Clinical Data

In this retrospective study, we included all consecutive EHOA patients with NP symptoms who underwent upper limb ENoG and nerve ultrasound and attended our EHOA Clinic at Padova University Hospital between January 2021 and December 2023. The exclusion criteria were neurological conditions, inflammatory rheumatic diseases, hand surgery within the last year, uncontrolled diabetes mellitus, uncontrolled hypertension, and active malignancies. This study was approved by the local Ethics Committee (approval number 446n/AO/23 on 12 February 2024), and written informed consent was obtained from all patients. The diagnosis of EHOA was defined as having at least one PIP or DIP with radiographic central erosion [1]. All patients included in this study had at least a gull wing deformity on X-rays, which is a characteristic sign of EHOA. The presence of NP was screened using the ID pain neuropathic pain-screening questionnaire (ID-Pain), which included 5 sensory items and one question asking if the pain is localized at the joint level with the aim of identifying nociceptive pain [15,16]. Patients with a score ≥ 1 (NP at least possible) on the ID-Pain questionnaire underwent ENoG and nerve ultrasound. In addition, all the following NP questionnaires were also used: (a) the DN4 questionnaire, which assesses pain symptoms and physical signs with a score ≥ 4 indicating NP [9,17], (b) the PainDETECT questionnaire, which includes 9 items: seven questions on the quality of pain, a descriptive question on the pattern of pain over time, and a question assessing radiating pain [10,18], and (c) the Italian version of the Neuropathic Pain Symptom Inventory (NPSI) [19]. The ID-Pain is a six-item self-questionnaire where the score ranges from −1 to 5, with higher scores indicating NP or mixed pain with a neuropathic component. Pain restricted solely to the joints (i.e., nociceptive pain) is scored as -1. The total score is obtained by summing the scores of the individual questions [15,16]. PainDETECT is a standardized 9-item questionnaire comprising seven questions related to the quality of pain, scored on a scale of 0–5, a descriptive question regarding the pain pattern over time, scored as −1/0/1, and a question evaluating radiating pain, scored as 0/2. Total scores are determined by adding up the individual question scores, resulting in a final score ranging from −1 to 38 [10,18]. The DN4 is a clinician-administered questionnaire comprising ten items: seven items focus on the quality of pain and are obtained by interviewing the patients, while the remaining three items are based on clinical examination and assess the presence or absence of touch or pinprick hypoesthesia and tactile allodynia. Each positive item is scored as 1, and each negative item is scored as 0. The total score is obtained by summing the scores of the individual questions [15,16,19]. The NPSI includes 12 items: ten descriptors of the different symptoms and two items for assessing the duration of spontaneous, ongoing, and paroxysmal pain. A total intensity score was calculated as the sum of the scores of the ten descriptors (0–10) and five subscores (0–4) [19]. Moreover, patients completed questionnaires aimed at assessing EHOA disease activity, namely the Australian/Canadian Osteoarthritis Hand Index (AUSCAN) and Dreiser’s algofunctional finger index [20,21]. The AUSCAN questionnaire, which scores from 0 (none) to 4 (extreme), comprises inquiries spanning three domains: pain, stiffness, and physical function. The total score is obtained by summing the scores of the 15 individual questions (0–60). In contrast, Dreiser’s algofunctional finger index evaluates functional status in patients with hand arthropathies, utilizing a 4-point scale to rate the patient’s capacity to perform daily tasks, where a score of 0 indicates no difficulty and a score of 3 indicates extreme difficulty. The total score is obtained by summing the scores of the 10 individual questions (0–30).

The following clinical and laboratory data were collected: age, sex, BMI, disease duration, intensity of pain (according to VAS between 0 and 10 on a Likert scale), painful and swollen joints, and inflammatory indices, as well as C-reactive protein (CRP) and erythrocyte sedimentation rate (ESR).

### 2.2. Assessment of Agreement between NP Screening Tools

The agreement among questionnaires was assessed for the following NP questionnaires: ID-Pain, DN4, and PainDETECT. For ID-Pain, the absence of NP was considered for scoring −1 and 0 = NP-improbable, while the presence of NP was considered for scoring between 1 and 5 = NP between possible and very probable. For the DN4 questionnaire, the presence of NP was considered for scores ≥ 4. For PainDETECT, the absence of NP was considered for values less than 13 (unlikely NP), while the presence was defined for values ≥13 (undetermined and likely NP).

### 2.3. Neurophysiologic Evaluation

Neurophysiological studies were performed according to the guidelines of the American Association of Electrodiagnostic Medicine, the American Academy of Neurology, and the American Academy of Physical Medicine and Rehabilitation by a neurologist with 35 years of experience in electromyography (MC) [22]. In detail, sensory nerve action potential (SNAP) amplitude and sensory conduction velocities (SCV) of the ulnar, median, and radial nerves, compound motor action potential (cMAP), motor conduction velocities (MCV), and distal motor latency (DML) of the median nerve were performed bilaterally. The cMAPs were evoked from the median nerves bilaterally (stimulation at the wrist and elbow; recording at the abductor pollicis brevis). For the assessment of potential CTS, the following neurophysiological studies of the median nerve were performed bilaterally: (1) SCV (orthodromic method) in four digit/wrist segments (the first, second, third, and fourth digits); (2) DML from the wrist to the thenar eminence.

Furthermore, when standard tests yielded normal results, comparative studies (median/ulnar comparison) were performed. According to Padua’s Scale, severity of CTS was classified into five classes [23] and was considered ‘negative CTS’ if normal findings were present in all tests (including comparative or segmental tests); ‘minimum CTS’: pathological findings only on segmental or comparative test; ‘mild CTS’: slowing of SCV in the median nerve (finger–wrist segment) and normality of DML; ‘moderate CTS’: slowing of sensory conduction of the median finger–wrist segment and abnormal DML; ‘severe CTS’: absence of at least one sensory response and abnormal DML; and ‘extreme CTS’: absence of both motor and sensory responses.

### 2.4. Ultrasound Evaluation

Ultrasound evaluation was performed as previously described [24] by an expert (MC) with 12 years of expertise in nerve US with a US system equipped with a high-frequency linear transducer, frequency range 10–18 MHz (MyLab Seven Esaote, Genova, Italy). The ultrasound examination of the median nerve at the wrist was considered a complementary study to the classic electroneurographic examination. The course of the median and ulnar nerves was followed bilaterally from the axilla to the wrist. The median nerve at the wrist was studied in the most proximal part of the carpal tunnel. The carpal tunnel inlet was defined as the proximal margin of the flexor retinaculum between the scaphoid tubercle and the pisiform bone, at the top of the flexor tendons of the fingers. By making small movements with the probe, the widest image of the nerve was sought, and at that point, the cross-sectional area (CSA) was measured. The appearance of the nerve can vary; sometimes it appears as a perfect ellipse, but sometimes its shape is irregular. It may be found to be bifid and rarely even trifid. The best visualized CSA was measured with the ‘ellipse method’ when applicable or the ‘tracing method’ when the nerve had an irregular shape. The mean CSA value of three measurements was considered. A CSA of <10 mm^2^ was considered normal.

### 2.5. Statistical Analysis

The data were expressed as medians and interquartile ranges. The McNemar test was employed to evaluate the concordance between questionnaires in assessing the presence of NP. To compare the characteristics of patients with and without NP, the Mann–Whitney test was used for continuous variables, whereas the Fisher’s exact test was used to compare categorical variables. The Spearman’s non-parametric test was used to analyze the correlations between the variables (GraphPad Prism version 6.0 for Windows, GraphPad Software, Boston, MA, USA, www.graphpad.com). A *p* value < 0.05 was considered significant. With regard to correlation coefficients, an r <0.3 was considered a negligible correlation, 0.3–0.5 was considered low correlation, 0.50–0.7 moderate, 0.71–0.9 high, and 0.91–1.00 very high.

## 3. Results

### 3.1. Neuropathic Pain Data

Of the 34 patients studied, 24 (70.6%) presented NP from possible to very likely according to the ID-Pain questionnaire. Patients with NP were statistically younger (65 vs. 75.5 years, *p* < 0.01) and had a higher VAS pain score compared to subjects without NP (6 [7–5] vs. 3.5 [4.75–2], *p* = 0.018). The scores of all the NP questionnaires utilized in this study exhibited statistically significant differences between the two groups (*p* < 0.01). No differences were observed between patients with and without NP regarding the following characteristics: painful and swollen joints, inflammatory markers, outcome measured, as well as the AUSCAN and Dreiser’s indices. The patients’ clinical and demographic data are outlined in Table 1.

Twenty-one (out of 24) patients underwent ENoG and median nerve ultrasound, whereas three patients declined instrumental examinations. According to DN4, 14 (41.2%) patients had NP; while using the PainDETECT questionnaire, 11 patients (32.4%) scored < 13 (unlikely NP), 11 patients (32.4%) scored between 13 and 18 (undetermined), and 12 patients (35.3%) scored > 18 (likely NP). The median score of NPSI in the whole population was 35 (49.3–20.8).

The 24 patients with NP (21 females) had a median age of 65 years (range 69.5–62.5) and a median BMI of 23.3 (27.1–21.4) kg/m^2^. The disease duration was 18 (22–12.5) years. The patients reported high levels of pain according to the VAS scale (median 6 [7–5]), with a median number of painful joints of 4 (7.3–2), and a median number of swollen joints of 0 (1–0). Inflammatory markers were normal: ESR 7 (11–5) mm/h and CRP 1.5 (2.9–0.8) mg/L. The median AUSCAN score was 36 (42.5–27.3), while Dreiser was 13.5 (16.3–6.8).

### 3.2. Agreement between NP Screening Tools

The McNemar test was conducted to determine the agreement between ID-Pain and PainDETECT in evaluating the presence/absence of NP. The McNemar test did not reveal a significant difference in the proportions of discordant pairs (*p* = 1), indicating strong agreement between ID-Pain and PainDETECT in determining the presence/absence of NP. All cases except one, classified as positive by one test, were also classified as positive by the other one, and similarly concordant data were detected for negative scores. These results suggest a high level of consistency and agreement between the two questionnaires for identifying patients with NP. Instead, the McNemar test revealed a significant difference in the proportions of discordant pairs between ID-Pain and DN4 (*p* = 0.0026) and between PainDETECT and DN4 (*p* = 0.004). Specifically, there were 11 discordant pairs (cases classified as positive by one test and negative by the other, or vice versa) between ID-Pain and DN4 and 10 discordant pairs between PainDETECT and DN4, indicating a lack of perfect agreement between the two tests. These results suggest a possible discrepancy in the classification of NP depending on the assessment method used, highlighting the importance of further investigation or validation of diagnostic procedures.

### 3.3. Electroneurography/Electromyography and Ultrasound Nerve Data

**Figure 1:**
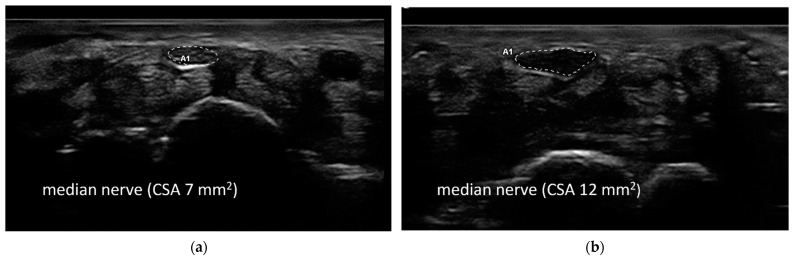
Axial ultrasonographic images of the median nerve at the wrist in a healthy control (**a**) (cross-sectional area 7 mm^2^) and in a patient with electrodiagnostically evaluated carpal tunnel syndrome (**b**) (cross-sectional area 12 mm^2^).

The ENoG was normal in 81% of patients, whereas the presence of CTS was detected in four patients: one right and three bilateral. The median CSA of the right median nerve at the wrist was 7 (8–7) mm^2^ and 6 (7–6) on the left side. The CSA was slightly increased (10–15 mm^2^) in one patient bilaterally, in two patients on the left side, and in one patient on the right side (Figure 1).

### 3.4. Correlations with Clinical Data

**Figure 2:**
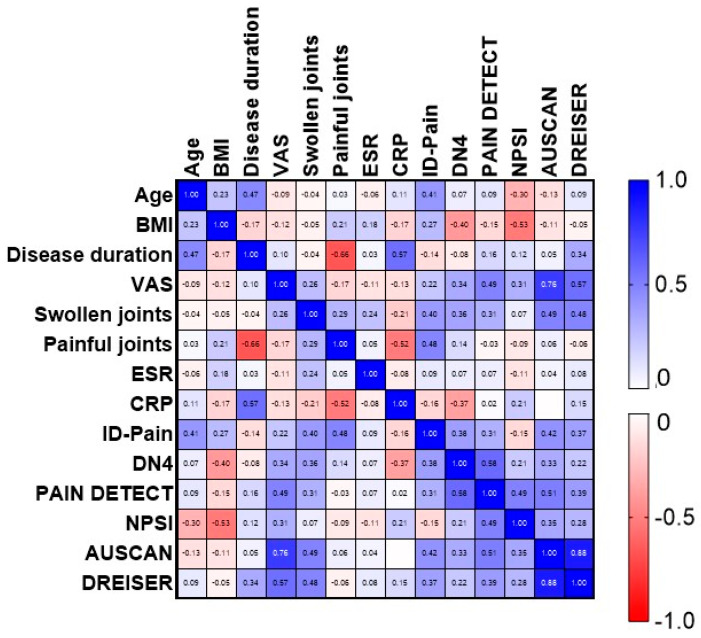
Correlation matrix. Negative correlations are displayed in red, while positive correlations are in blue. BMI = body max index; VAS = visual analog scale; ESR = erythrocyte sedimentation rate; CRP = C-reactive protein; ID-Pain = ID pain neuropathic pain-screening questionnaire; DN4 = Douleur Neuropathique en 4 Questions; NPSI = Neuropathic Pain Symptom Inventory; AUSCAN = Australian/Canadian Osteoarthritis Hand Index; Dreiser = Dreiser’s algofunctional finger index.

Disease duration correlated moderately with age (r = 0.47, *p* = 0.04) and CRP (r = 0.57, *p* = 0.01) (Figure 2). The number of painful joints correlated negatively with CRP (r = −0.52, *p* = 0.02) and disease duration (r = −0.66, *p* = 0.001). There was a slight positive correlation between the number of swollen joints and AUSCAN and Dreiser’s scores (r = 0.49, *p* = 0.02, r = 0.48, *p* = 0.03, respectively).

The ID-Pain questionnaire correlated with the number of painful joints (r = 0.48, *p* = 0.03) and with the AUSCAN questionnaire (r = 0.37, *p* = 0.05). The DN4 questionnaire correlated with PainDETECT (r = 0.58, *p* < 0.01). The PainDETECT questionnaire correlated with VAS pain (r = 0.49, *p* = 0.02), the DN4 questionnaire (r = 0.58, *p* < 0.01), and AUSCAN (r = 0.51, *p* = 0.02). The NPSI questionnaire correlated negatively with BMI (r = −0.53, *p* = 0.01) and positively with the PainDETECT questionnaire (r = 0.49, *p* = 0.02).

## 4. Discussion

In the present study, we explored the presence and characteristics of NP in EHOA. Our findings revealed that 32–70% of EHOA patients exhibited symptoms consistent with NP, variable according to the assessment questionnaire. Surprisingly, despite patients frequently exhibiting symptoms compatible with NP, only 19% of patients presented alterations on ENoG and ultrasound examinations confirming CTS. This suggests a probable nociplastic component for pain in patients with EHOA, which warrants tailored treatment. In the present study, NP was correlated with clinical and functional indices of EHOA.

Pain in OA is a complex phenomenon involving nociceptive signals originating from joint tissue damage and neuronal sensitization in both the peripheral and central nervous systems. The interactions between these diverse components vary in each subject, evolve over time, and depend on the disease stages, which can exacerbate painful symptoms [25]. Additionally, it should be noted that in OA, pain does not always correlate with structural damage [26]. There has been growing interest in NP more recently, resulting in a rise in the number of available publications, particularly on knee OA. Few studies to date have been published on NP in hand OA, and there is scarce evidence; no study has specifically investigated the frequency and characteristics of NP in EHOA. The study by Van der Meulen et al. [10] found that 73% of subjects had EHOA and the presence of erosions did not correlate with NP; however, no specific analyses were performed to ascertain whether NP differed between erosive and non-erosive forms. In the study by Pettersen et al., patients with EHOA comprised 35% (102/291) of patients, and they had a lower pressure pain threshold at the wrist and tibialis anterior and greater temporal summation; nevertheless, the authors concluded that the differences were small and suggested that central sensitization was mainly explained by other factors than joint pathology [27].

We used the ID-Pain questionnaire as a screening tool to assess the presence of NP [28]. Importantly, this questionnaire includes an item that assesses whether neuropathic symptoms are localized at the joint level, taking into account the presence of nociceptive pain. We also used two other questionnaires: DN4 and PainDETECT, as well as NPSI, which was specifically designed to evaluate NP. A previous study used the DN4 questionnaire and found that 42% of patients with hand OA experienced NP [9], in line with our own findings using the same questionnaire. Another study previously used the PainDETECT questionnaire and reported an NP frequency of 19% in patients with hand OA [10], which is lower than that observed in our study (32.4%) using this questionnaire. Petterson et al. found a prevalence of 42% for central sensitization measured with temporal summation tests in patients with hand OA [27]. Considering that the frequency of neuropathic pain varies depending on the questionnaire used, it would be beneficial to validate a combination of questionnaires as a future screening tool to assess the presence of NP.

We performed an agreement analysis between questionnaires, which demonstrated consistency in assessing the presence/absence of NP between ID-Pain and PainDETECT but not between the former two and DN4. Nevertheless, the DN4 correlated positively with PainDETECT questionnaires at the correlation analysis, and generally speaking, all questionnaires were statistically different between the groups of patients with and without NP.

In the literature, DN4 and ID-Pain have been demonstrated to be highly interrelated and comparable in identifying NP [28]. One possible explanation for our conflicting results may be attributable to the fact that DN4 involves a clinical assessment, which in our case was performed by a rheumatologist rather than a neurologist. This could have influenced the accuracy of physical examination findings, potentially resulting in either an underestimation or an overestimation of NP signs [15,16]. Furthermore, DN4 involves a dichotomous assessment, where symptoms are categorized as either indicative or non-indicative of NP, whereas ID-Pain and PainDETECT consider different degrees of probability of having symptoms compatible with NP (i.e., improbable, possible, probable, and very probable for ID-Pain; and unlikely, indeterminate, and likely for PainDETECT) [15,16].

The EHOA is a particularly inflammatory and more aggressive form of hand OA [1]. Synovial inflammation—with histological [1] or ultrasound findings of hypertrophy and increased power doppler signal or MRI—is more pronounced than in the non-erosive form and is associated with joint damage [29,30]. The role of inflammation is also confirmed by the overexpression of inflammatory cytokines, such as soluble interleukin 2 receptor and myeloperoxidase, in the serum of patients affected by EHOA vs. controls [1,31]. Unfortunately, no EHOA studies to date have evaluated the expression of neuropeptides, such as nerve growth factor or calcitonin gene-related peptide, which could be involved in neuroinflammation and therefore in NP.

Despite patients frequently having symptoms compatible with NP, we found that only 19% of patients presented alterations on ENoG and ultrasound examinations confirming CTS. Since most patients showed no evidence of structural damage to the nervous system lesions on ENoG, the abnormalities detected by neuropathic-like or better non-nociceptive pain questionnaires might have a nociplastic etiology indicative of alterations in the nervous system without apparent tissue damage, thus suggesting a central sensitization component [32].

In our study, the number of painful joints correlated positively with AUSCAN and Dreiser’s scores. Additionally, the number of painful joints correlated negatively with disease duration, bearing in mind that our study population consisted of a long-standing cohort of EHOA patients with a median disease duration of 18 (22–12.5) years. These findings reflect the clinical observation that the inflammatory phase evident in the early stages of the disease might decrease over time. We also found that patients who exhibited NP were younger and had higher VAS, confirming previous reports in the literature [10,27] and highlighting the need to target NP-like pain other than nociceptive pain. Indeed, the NP questionnaires correlated with clinical outcomes: the ID-Pain correlated with the AUSCAN, whereas the PainDETECT correlated with both the VAS pain and the AUSCAN. In the Van der Meulen et al. study [10], the steroid treatment did not alleviate NP, indicating that patient-tailored treatment may be necessary for non-nociceptive pain. In fact, in a prospective randomized clinical study, 65 patients with hand OA were treated with duloxetine, pregabalin, or placebo [33]. The pregabalin group demonstrated improvement in pain, AUSCAN pain, and AUSCAN function. No differences were found between duloxetine and placebo [33]. In OA pathology, the following different phenotypes are recognized: metabolic, inflammatory, post-traumatic, and osteoporotic. Additionally, different OA pain phenotypes are recognized: nociceptive, neuropathic, and nociplastic [34]. Pain in OA does not necessarily correlate with structural damage. In the cohort, not all patients presented symptoms suggesting neuropathic pain as well as burning or tingling. For this reason, in clinical practice, it is important to evaluate the presence of different types of pain in order to utilize target treatments for chronic pain (anti-inflammatory drugs, anti-neutrophic factors, antidepressants, or anticonvulsants) [35,36]. Furthermore, in clinical trials, the phenotyping of patients into specific subgroups is necessary to assess the efficacy of treatments in specific subsets of patients. Additionally, in cases of neuropathic symptoms, it is important to perform an electromyography to exclude carpal tunnel syndrome, which may be susceptible to surgical intervention.

We would be remiss not to mention some of the limitations of our study. Despite the small sample size, we were able to assess the presence and characteristics of NP in patients affected by EHOA from both clinical and instrumental standpoints. Furthermore, we used and compared the effectiveness of different screening tools to evaluate the presence of NP. In addition, ENoG was performed to evaluate the presence of CTS, which is known to cause NP symptoms. Finally, the presence of NP correlated with hand OA clinical indices. Other limitations of this study include its retrospective and single-center design, the lack of an erosion radiographic scoring system, and the absence of a control group.

## 5. Conclusions

Our study revealed that 32% to 70% of EHOA patients exhibited symptoms consistent with NP, with observed variability depending on the questionnaire utilized for the assessment. The ID-Pain and PainDETECT questionnaires were consistent in determining the presence of NP. Such agreement was not found between the DN4 questionnaire and the other NP questionnaires. Despite patients frequently exhibiting symptoms compatible with NP, only 19% of patients presented alterations on ENoG and ultrasound examinations confirming CTS. This suggests a probable nociplastic component for pain in patients with EHOA, which warrants tailored treatment. In the present study, NP correlated with clinical and functional indices of EHOA.

## Figures and Tables

**Table 1:** Patients’ demographic and clinical features.

Features	Not NP (*n* = 10)	NP (*n* = 24)	*p*
Women	8 (80%)	21 (87.5%)	0.62
Median age (IQR) years	75.5 (76–72)	65 (69.5–62.5)	0.007 *
Median body mass index (BMI) (IQR) kg/m^2^	25 (25.5–25.4)	23.3 (27.1–21.4)	0.87
Median disease duration (IQR) years	17.5 (22.5–13)	18 (22–12.5)	0.57
Median VAS (IQR) (0–10)	3.5 (4.75–2)	6 (7–5)	0.018 *
Median swollen joints (IQR) (number)	0 (0.75–0)	0 (1–0)	0.81
Median painful joints (IQR) (number)	3 (4–1.25)	4 (7.3–2)	0.30
Median erythrocyte sedimentation rate (IQR) mm/h	10.5 (15.3–3)	7 (11–5)	0.70
Median C-reactive protein (IQR) mg/L	2.9 (2.9–0.9)	1.5 (2.9–0.8)	0.68
Median AUSCAN (IQR) (0–60 scale)	29 (30–28)	36 (42.5–27.3)	0.07
Median Dresier (IQR) (0–30 scale)	8 (9.8–8)	13.5 (16.3–6.8)	0.31
Median ID-Pain (IQR)	0 (0–0)	2 (3–1)	<0.001 **
Median DN4 (IQR)	0.5 (1–1)	3.5 (4–2)	<0.001 **
Median PainDETECT (IQR)	7 (10–7)	18.5 (23.3–16.5)	<0.001 **
Median NPSI (IQR)	14 (28–13)	48 (54.3–35)	<0.001 **

BMI = body max index; IQR = interquartile range; VAS = visual analog scale; AUSCAN = Australian/Canadian Osteoarthritis Hand Index; Dreiser = Dreiser’s algofunctional finger index; ID-Pain = ID pain neuropathic pain-screening questionnaire; DN4 = Douleur Neuropathique en 4 Questions; NPSI = Neuropathic Pain Symptom Inventory; * *p* < 0.05, ** *p* < 0.01.

## Data Availability

The data that support the findings of this study are available from the corresponding author, R.R., upon reasonable request.
